# Harvard Veterinary Department’s Peril

**Published:** 1900-12

**Authors:** 


### HARVARD VETERINARY DEPARTMENT'S PERIL.

THE college year of 1900–01 had hardly reached its first quarter mark when the news reached us of a determination of the Board of Governors of Harvard College to close the Department of Veterinary Medicine. Such a decision at this time seems almost beyond comprehension, for with the better conditions prevailing in the animal industry world there has followed an exceptional increase in the classes of every school in North America. With the field of the automobile well defined and the higher and better place of the horse fixed, it would seem, indeed, a strange period for such a decision. With the veterinary sanitary control work of our land extending over a wider and better field, and the unsupplied demand of our government for more assistance, there should have been no time so auspicious as the opening college year, for the past ten years at least. But such is the decision, unless there shall be given substantial aid in the way of endowing this department.

After many years of earnest college work, the growth of an alumni body of considerable size, this department, whose help and aid should have filled many a breach, and whose strong arm of support should have contributed money sufficient to carry on the good work, there would seem to be little appreciation of the work of the veterinarian in the New England States when such an institution fails to receive sufficient of an endowment fund to perpetuate its work. With large and varied interests in the animal world, from sentiment in humane societies, from sport in the racing world, from pleasure in the driving classes, from the health seekers among the riding classes, from the wealth seekers in the industrial fields of the employment of horses, from the value of protective measures in the food-supply derived from animals, for the safeguards vouchsafed by veterinarians in the inspection of animals subject to diseases transmissible to man, and in many other ways enriching and ennobling a community, there would seem an utter forgetfulness of the value of such an institution. If it cannot grow stronger and better; if it cannot keep pace with the advancement of laboratory work and special fields of investigation ; if its sphere of usefulness in higher education has reached its zenith, and there are no greater victories to achieve, then it would seem wise to close its doors rather than die by starvation.

We sincerely trust that the wealth of the New England States will not be so wholly forgetful of the grave responsibilities wealth incurs as to leave this college without sustenance at a time when many are contemplating the creation of new institutions, while well-equipped ones exist without the necessary means of maintenance. Wake up, down East! and save this institution, and let no such blot of shame rest upon you as a broad, liberal, and educated community of people.

